# Predictive value of hematological indices on incidence and severity of pulmonary embolism in COVID‐19 patients

**DOI:** 10.1002/iid3.1012

**Published:** 2023-09-29

**Authors:** Hanieh Radkhah, Ensieh Sadat Mansouri, Shiva Rahimipour Anaraki, Milad Gholizadeh Mesgarha, Ali Sheikhy, Mohamad Mehdi Khadembashiri, Mohamad Amin Khadembashiri, Mohamad Eslami, Tara Mahmoodi, Behnaz Inanloo, Arash Pour Mohammad

**Affiliations:** ^1^ Department of Internal Medicine, School of Medicine, Sina Hospital Tehran University of Medical Sciences (TUMS) Tehran Iran; ^2^ Faculty of Medicine Iran University of Medical Sciences (IUMS) Tehran Iran; ^3^ Students' Scientific Research Center Tehran University of Medical Sciences (TUMS) Tehran Iran; ^4^ Sina Hospital Tehran University of Medical Sciences (TUMS) Tehran Iran

**Keywords:** neutrophil‐to‐lymphocyte ratio, PESI score, platelet‐to‐lymphocyte ratio, pulmonary thromboembolism, SARS‐CoV‐2

## Abstract

**Background:**

Pulmonary thromboembolism (PTE) is a common complication of severe acute respiratory syndrome coronavirus 2 (SARS‐CoV‐2), which raises the COVID‐19 disease's fatality rate from 3% to 45%. Nevertheless, due to fairly indistinguishable clinical symptoms and a lack of validated clinical prediction models, PTE diagnosis in COVID‐19 patients is challenging. This study aims to investigate the applicability of hematological indices to predict PTE incidence and its severity in SARS‐CoV‐2 patients.

**Methods:**

A retrospective cohort study was conducted on hospitalized patients with a confirmed diagnosis of SARS‐CoV‐2 infection who underwent CT angiography to assess probable PTE in them. The correlation between complete blood count parameters 1 day before CT angiography and CT angiography outcomes, and simplified pulmonary embolism severity index (s‐PESI) was investigated.

**Results:**

We discovered that among individuals with a probable PTE, males and those with higher platelet‐to‐lymphocyte (PLR) and neutrophil‐to‐lymphocyte (NLR) ratios had a greater likelihood of PTE incidence (*p* < .001, .027, and .037, respectively). PLR was a significant and independent predictor of PTE with a *p* value of .045. Moreover, a higher neutrophil count was associated with a higher s‐PESI score in COVID‐19 patients developing PTE (*p*: .038).

**Conclusions:**

Among hematological indices, NLR and more precisely PLR are cost‐effective and simply calculable markers that can assist physicians in determining whether or not COVID‐19 patients with clinically probable PTE require CT angiography and the higher neutrophil count can be employed as an indicator of PTE severity in COVID‐19 patients. Further large multicenter and prospective studies are warranted to corroborate these observations.

## 1 INTRODUCTION

The severe acute respiratory syndrome coronavirus 2 (SARS‐CoV‐2) outbreak that began in Wuhan, China, in December 2019 rapidly spread worldwide, insofar as the World Health Organization has declared COVID‐19 to be a pandemic and a public health emergency of international concern.^1^, ^2^, ^3^, ^4^ Although the disease is typically associated with respiratory tract involvement, the virus also can affect other systems including hematological and cardiovascular systems through different hypothesized mechanisms.^5^, ^6^ One of the most important underlying mechanisms is an increased level of inflammatory mediators.^7^ This inflammatory process is accompanied by thrombotic events, including pulmonary thromboembolism (PTE), in COVID‐19 patients.^8^, ^9^, ^10^

PTE is a cause of mortality among COVID‐19 complications. While the case fatality rate for COVID‐19 ranges from 2% to 3%, the mortality rate for COVID‐19 patients who develop PTE is 45.1%.^11^ This remarkable complication is reported in 15.3% of COVID‐19 cases.^12^ Due to the high mortality and incidence of PTE, as well as the fact that prompt treatment is highly effective and has been shown to significantly impact clinical outcomes, early diagnosis of PTE is crucial.^12^, ^13^

Nevertheless, PTE diagnosis in COVID‐19 is challenging for a variety of reasons. First, PTE and COVID‐19 clinical manifestations may overlap.^14^ Second, hospital overcrowding during the resurgence of the COVID‐19 epidemic, costs, and lack of availability in all centers make chest CT angiography (CTA) inaccessible for all patients with probable PTE.^15^ Third, available clinical prediction models for PTE do not apply to COVID‐19 patients. Therefore, their use as the sole diagnostic screening tool in clinical practice is not recommended. New clinical probability models for PTE that have been validated in COVID‐19 patients are consequently required.^16^

As previously stated, thrombotic events accompany the inflammatory process in patients with COVID‐19.^8^, ^9^, ^10^ Notably, hematological ratios are one of the valuable, inexpensive, and widely‐examined markers of inflammation.^17^ In addition, circulating biomarkers of inflammation, such as neutrophil‐to‐lymphocyte ratio (NLR) and platelet‐to‐lymphocyte ratio (PLR), have been proposed as reliable prognosticators for both COVID‐19 and PTE patients.^18^, ^19^, ^20^ Despite the importance of the aforementioned hematologic markers, very few studies focus on predicting PTE occurrence or PTE severity in COVID‐19 patients with probable PTE. The results of these studies were somewhat contradictory to each other; while one study's results showed there was no correlation found between NLR and PLR levels and PTE development,^21^ another study indicated that higher NLR, PLR at admission strongly predict acute PTE risk in COVID‐19 patients.^22^

According to this lack of enough evidence, we conducted an exploratory analysis to evaluate the potential role of these parameters as predictors of thromboembolism in COVID‐19 patients and the correlation of complete blood count (CBC) parameters with the simplified‐ pulmonary embolism severity index (s‐PESI) score as an indicator of PTE severity.

## 2 METHOD

### 2.1 Participants and study design

On the routine basis in the hospital, COVID‐19 patients with probable PTE due to changes in their clinical condition who were patients with poor response to treatment, hemoptysis, and spontaneous deterioration, comprising sudden respiratory distress, tachycardia, sudden drop in blood pressure, or O_2_ saturation, underwent Chest CTA immediately after diagnostic doubt.^23^ All these patients received prophylactic anticoagulant and every patient who was diagnosed with PTE was treated by the standard protocol of PTE treatment. Among these above‐mentioned patients, cases with a confirmed diagnosis of SARS‐CoV‐2 infection based on positive chest CT scan findings or positive nasopharyngeal reverse transcriptase‐polymerase chain reaction test at the general ward and ICU of Sina Hospital, Tehran, Iran, between “March 2019” and “July 2022,” were enrolled in this observational, analytical, retrospective cohort study and based on the results of CTA, they were divided into the PTE and non‐PTE groups.

Subsequently, patients meeting exclusion criteria consist of (1) Previous history of deep vein thrombosis or PTE, (2) Severe sepsis, (3) End‐stage renal disease, (4) Cirrhosis, (5) Hemoglobinopathies (e.g., Thalassemia), (6) Platelet disorders (e.g., Immune thrombocytopenia, Thrombotic thrombocytopenic purpura), (7) Hematological malignancies (e.g., acute lymphoblastic leukemia, chronic lymphocytic leukemia, lymphoma), 8. Immunosuppressive drugs use, and (9) Blood products transfusion within 48 h of blood sampling were excluded, and the remaining patients were included in the final sample.

### 2.2 Data collection

COVID‐19 patients data were collected during 2 years of the pandemic in Iran which encompassed six peaks of disease and various subtypes of SARS‐CoV‐2 infections. Patients' blood samples were checked for CBC (including white blood cell [WBC] count and differentials Neutrophil and Lymphocyte], platelets count, Hemoglobin, red cell distribution width, mean platelet volume [MPV]), d‐dimer, C‐reactive protein, and ferritin, then NLR and PLR calculated and analysis was performed on the laboratory results of 24 h before CTA. Moreover, the s‐PESI score was estimated for the patients with confirmed PTE on the day of its diagnosis.

### 2.3 Statistical analysis

Statistical analysis was done on the aforementioned laboratory data, simplified PESI score in addition to patients' age and gender in each PTE and non‐PTE group. All the collected data were entered into IBM SPSS Statistics for Windows, version 22.0 (IBM Corp.) and R version 4.0.3 for analysis.

Qualitative data were described as absolute frequencies and percentages, and quantitative data were reported as mean and standard deviation or median and interquartile range according to their distribution. The normality of the variables was assessed using histogram charts as well as central tendency and dispersion measures. The qualitative variables were compared between the “non‐PTE” and “PTE” groups applying the chi‐square test, and the quantitative variables were compared between the two above‐mentioned groups using *t*‐test for normally distributed and Mann–Whitney U test for skewed distributed variables.

The univariate effect of covariates on PTE was assessed by the “Logistic regression” model and reported as an odds ratio (OR) with 95% confidence interval. Covariates with *p* < .1 in the univariate Logistic regression analyses were entered into multivariate Logistic regression analysis. The backward elimination method was considered for multivariate Logistic regression analysis to locate predictors of PTE.

Receiver operator characteristic (ROC) curves were generated to estimate the accuracy of each covariate in predicting PTE. The area under each ROC curve (AUC) was calculated to identify an accurate prognostic covariate.

## 3 RESULT

### 3.1 Study population

Among 464 COVID‐19 patients who underwent CTA, 28 patients were excluded from the study according to inclusion and exclusion criteria. Of these excluded patients, nine patients were diagnosed with PTE. The demographic and characteristics of included patients are shown in Table 1. In brief, male gender were significantly correlated with PTE occurrence in patients with underlying COVID‐19 (81.7% vs. 41.6%, *p* < .001). The median age had no significant differences between two groups. Except for NLR and PLR, other lab data including MPV had no significant differences between two studied groups. NLR was significantly higher in PTE group (235.50 vs. 205.50, *p* value: .027). Comparably, PLR was significantly higher in COVID patients which led to PTE (13.65 vs. 9.94, *p* value: .037).

**Table 1:** Patients' baseline characteristics and lab data.

	No‐PTE (*N* = 250)	PTE (*N* = 186)	*p* Value
Sex			
Male	104 (41.6%)	152 (81.7%)	<.001
Female	146 (58.4%)	34 (18.3%)	
Age	55 [54–57]	57 [54–59]	.842
Hemoglobin	12.71 ± 0.35	12.63 ± 0.17	.821
Platelet	257.41 ± 6.88	256.05 ± 8.40	.900
WBC	11.76 ± 0.48	10.01 ± 1.01	.197
Neutrophil %	78.34 ± 0.77	80.43 ± 0.92	.083
Lymphocyte %	14.00 ± 0.59	12.30 ± 0.65	.063
RDW	14.84 ± 0.14	14.60 ± 0.20	.539
MPV	10.72 ± 0.26	10.44 ± 0.08	.408
Ferritin	523.40 [455.90–623.69]	588.15 [496.20–736.00]	.460
d‐dimer	763.00 [681.00–952.00]	1059.00 [780.00–1471.00]	.106
CRP	37.40 [30.90–51.30]	36.20 [28.10–48.00]	.712
PLR	205.50 [183.00–222.00]	235.50 [198.00–295.11]	**.027**
NLR	9.94 ± 0.93	13.65 ± 1.70	**.037**
SII	113.94 [96.87–135.38]	124.32 [112.98–145.44]	.931

*Note*: Bold values are statistically significant *p* < 0.05.

Abbreviations: CRP, C‐reactive protein; MPV, mean platelet volume; NLR, neutrophil/lymphocyte ratio; PLR, platelet/lymphocyte ratio; PTE, pulmonary thromboembolism; RDW, red cell distribution width; SII, Systemic Inflammatory Immune Index; WBC, white blood cell.

### 3.2 Stepwise logistic regression analyses of the disease determinants

All variables with a *p* ≤ .1 in univariate analyses (Table 2) were included in stepwise logistic regression (Table 3). According to the univariate analysis, patients with higher Neutrophil percentage, PLR, and NLR 24 h before CTA had a higher risk of PTE incidence; however, they were not statistically significant except PLR. The OR was estimated as 1.01 (0.99–1.03) for Neutrophil percentage, 1.01 (1.00–1.02) for PLR, and 1.01 (1.00–1.02) for NLR. In contrast, patients with higher lymphocyte percentage had a lower chance of PTE although it was not statistically significant. The OR for mentioned protective factor was estimated as 0.98 (0.96–1.00). In the multivariate analysis, PLR and NLR 24 h before CTA were revealed as potential determinants of PTE (OR: 1.001 (1.000–1.002) and OR: 1.006 (0.998–1.018), respectively).

**Table 2:** Univariate models for PTE: OR and 95% 95% CI.

Variable	OR	95% CI	*p* Value
WBC	1.00	0.99–1.01	.552
Neutrophil percentage	1.01	0.99–1.03	.092
Lymphocyte percentage	0.98	0.96–1.00	.065
Hemoglobin	0.99	0.96–1.04	.852
RDW	0.98	0.90–1.05	.526
MPV	0.96	0.86–1.08	.489
Platelet count	1.00	0.99–1.00	.901
PLR	1.01	1.00–1.02	**.008**
NLR	1.01	1.00–1.02	.062
CRP	0.99	0.99–1.00	.711
Ferritin	1.00	0.99–1.01	.492
SII (×10^9^/L)	1.00	0.99–1.01	.921

*Note*: Bold value statistically significant *p* < 0.05.

Abbreviations: CI, confidence intervals; CRP, C‐reactive protein; MPV, mean platelet volume; NLR, neutrophil/lymphocyte ratio; OR, odds ratios; PTE, pulmonary thromboembolism; PLR, platelet/lymphocyte ratio; RDW, red cell distribution width; SII, Systemic Inflammatory Immune Index; WBC, white blood cell.

**Table 3:** Final model for PTE—results from stepwise logistic regression.

	OR	95% CI	*p* Value
PLR	1.001	1.000–1.002	**.045**
NLR	1.006	0.998–1.018	.053

*Note*: Variables are listed in the order of inclusion into the model. Bold value statistically significant *p* < 0.05.

Abbreviations: CI, confidence intervals; NLR, neutrophil/lymphocyte ratio; OR, odds ratios; PTE, pulmonary thromboembolism; PLR, platelet/lymphocyte ratio.

### 3.3 ROC curve analysis

**Figure 1:**
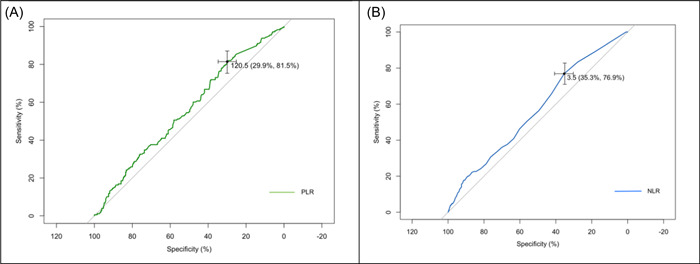
The receiver operator characteristic curves of pulmonary thromboembolism determinants (A) platelet/lymphocyte ratio and (B) neutrophil/lymphocyte ratio.

The performance of PLR and NLR is shown in Figure 1. Discrimination at the PTE was poor overall (0.5 < AUC < 0.6), although there were significant indicators. The area under the curve for PLR was estimated as 0.559 (*p* value: .001) and for NLR as 0.567 (*p* < .001). The threshold for PLR was estimated as 120.5 as the best cut‐off point, moreover, the ideal cut‐off for the NLR was estimated as 3.5 (Table 4).

**Table 4:** ROC analysis of predictive variables.

Covariate	AUC/*p* value	Thresholds	Specificity	Sensitivity
PLR	0.559/.001	120.5	29.94% (25.15%–34.73%)	81.46% (75.28%–87.08%)
NLR	0.567/<.001	3.5	35.33% (29.94%–40.42%)	76.88% (70.97%–82.80%)

Abbreviations: AUC, area under each ROC curve; NLR, neutrophil/lymphocyte ratio; PLR, platelet/lymphocyte ratio; ROC, receiver operator characteristic.

### 3.4 Linear regression analyses for simplified PESI‐score determinants in PTE patients

**Figure 2:**
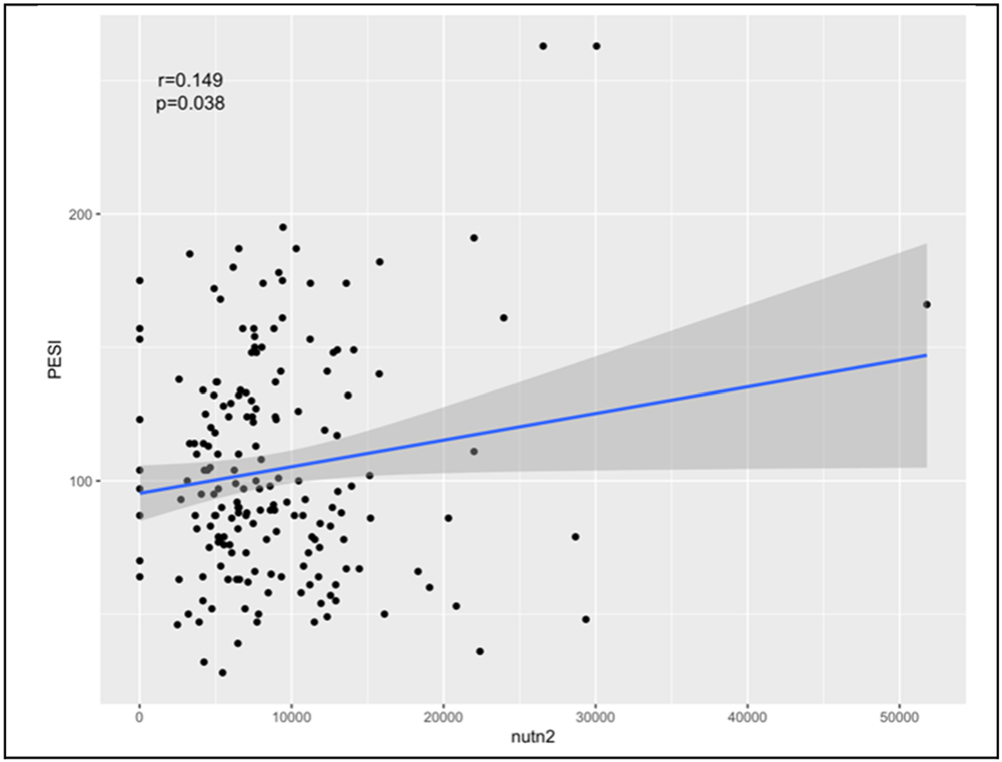
Correlation between simplified pulmonary embolism severity index score and neutrophil count.

In the linear regression analysis, higher neutrophil count in 24 h before CTA was identified as a potential indicator of PTE severity based on the simplified PESI‐score (Beta coefficient: 0.149, *p* value: .038) (Table 5) (Figure 2).

**Table 5:** Linear regression analyses for PESI score in PTE patients.

	Unstandardized coefficients		Standardized coefficients (β)	*P* Value	95% CI
	B	SE			
WBC	−0.001	0.003	−0.027	.721	−0.006, 0.004
Neutrophil percentage	0.453	0.259	0.131	.082	−0.058, 0.964
Lymphocyte percentage	−0.321	0.365	−0.066	.380	−1.041, 0.399
Neutrophil count	0.001	0.001	0.149	**.038**	0.001, 0.003
Lymphocyte count	−0.003	0.004	−0.063	.396	−0.010, 0.004
Hemoglobulin	−1.786	1.383	−0.097	.198	−4.515, 0.943
RDW	1.331	1.210	0.083	.273	−1.057, 3.719
MPV	−2.974	2.839	−0.079	.296	−8.576, 2.628
Platelet count	−0.029	0.029	−0.077	.311	−0.086, 0.027
PLR	−0.007	0.013	−0.042	.579	−0.032, 0.018
NLR	−0.004	0.133	−0.002	.978	−0.276, 0.259
CRP	0.043	0.068	0.053	.525	−0.091, 0.177
Ferritin	0.005	0.008	0.057	.563	−0.011, 0.021
SII (×10^9^/L)	0.001	0.014	0.006	.940	−0.027, 0.029

*Note*: Bold value statistically significant *p* < 0.05.

Abbreviations: CRP, C‐reactive protein; MPV, mean platelet volume; NLR, neutrophil/lymphocyte ratio; PLR, platelet/lymphocyte ratio; RDW, red cell distribution width; SII, Systemic Inflammatory Immune Index; WBC, white blood cell.

## 4 DISCUSSION

Our study indicated that PLR significantly and independently predicts PTE occurrence in hospitalized COVID‐19 patients. Furthermore, the bivariate test revealed an association between NLR and PTE incidence in these patients, notwithstanding regression analysis revealed that the correlation was not independently significant. Additionally, a greater neutrophil count was shown to be a marker that is associated with a higher PTE severity (higher s‐PESI score).

### 4.1 NLR and PLR predictive value in COVID‐19 infection and its pulmonary embolism complication

A systematic review and meta‐analysis demonstrated that NLR has adequate predictive values on disease severity and mortality in patients developing COVID‐19 infection. In severe or nonsurvival patients with COVID‐19, the lymphocytes count decreases progressively, while the neutrophils count gradually increases.^18^ This may be due to excessive inflammation and immune suppression caused by SARS‐CoV‐2 infection. On the one hand, neutrophils are generally regarded as proinflammatory cells with a range of antimicrobial activities, which can be triggered by virus‐related inflammatory factors, such as interleukin‐6 and interleukin‐8. On the other hand, systematic inflammation triggered by SARS‐CoV‐2 significantly depresses cellular immunity, leading to a decrease in CD3 + T cells, CD4 + T cells, and CD8 + T cells. In addition, SARS‐CoV‐2‐infected T cells may also cause cytopathic effects on T cells. Therefore, the ratio of neutrophils to lymphocytes increases.^18^, ^24^ Even though the etiopathogenesis of PTE in COVID‐19 is not completely understood, factors related to the acute inflammatory response to the disease may be contributing to a dysregulation of the equilibrium of procoagulant and anticoagulant mechanisms. Accordingly, it could reasonably be predicted that higher NLR values were also found in COVID‐19 patients with PTE when compared to patients with non‐PTE. A single‐center prospective cohort study by Castillejo et al. indicated higher baseline, peak, and prior‐to‐CTPA NLR values in PTE groups which were statistically significant compared to the non‐PTE group.^14^ This observation was consistent with our bivariate analysis of NLR and PTE incidence; however, regression analysis did not show this significance.

A systematic review and meta‐analysis which investigated the prognostic role of PLR in COVID‐19 noted that a higher level of PLR on admission in COVID‐19 patients is correlated with increased morbidity and mortality but evidence regarding this issue has low quality,^25^ but another systematic review and meta‐analysis focusing on the association between hematological indices and COVID‐19 progression and mortality found that PLR had no significant correlation with progression and fatality of the disease.^26^ Based on this review of the literature, it can be concluded that NLR is better at predicting the severity and mortality of COVID‐19 disease than PLR.^27^ On the contrary, our study showed PLR but not NLR significantly and independently predicts PTE incidence in hospitalized COVID‐19 patients. PLR was shown to be positively correlated with CT pulmonary artery obstruction index which suggests that higher PLR is associated with escalated thrombus burden.^28^ Consequently, we could postulate that for thrombotic complications of COVID‐19 like PTE, PLR has a more precise predictive value.

Considering the cut‐off point for the predictive power of NLR and PLR, ROC curve analysis in our study showed a threshold of 3.5 for NLR with 81.4% sensitivity and 29.9% specificity and a threshold of 120.5 for PLR with 76.8% sensitivity and 35.3% specificity. Another observational, analytical, retrospective cohort study demonstrated a threshold of 13.67 for NLR with 67.7% sensitivity and 81% specificity and a threshold of 207.06 with 74.2% sensitivity and 61.3% specificity to predict the occurrence of acute PTE in COVID‐19 patients.^22^ The difference between our cut‐off values and the study by Muresan et al. can be ascribed to the various time of laboratory data acquisition. In our study, we utilized laboratory results 24 h before CTA but Muresan et al. used their first laboratory analyses. Nonetheless, it may be concluded from these results that earlier NLR and PLR calculations, with fairly acceptable specificity for NLR and fairly acceptable sensitivity for PLR in the COVID‐19 disease course, could predict the incidence of PTE with higher cut‐off values; in comparison, later NLR and PLR calculation could also predict PTE incidence with to some extent acceptable sensitivity but very low specificity with lower cut‐off values.

In view of the comparison between the predictive role of NLR and PLR for venous thromboembolism (VTE) in COVID‐19 patients versus non‐COVID‐19 patients, numerous studies have investigated the role of NLR and PLR in the prediction of VTE probability and mortality in different patients population.^29^ Considering the NLR, Farah et al. found a cut‐off value of 5.3 for NLR with an AUC of 0.67, a sensitivity of 69%, and a specificity of 57% in a study population with acute VTE patients versus non‐VTE patients. With an approximately similar AUC, they obtained a higher cut‐off for NLR with lower sensitivity and higher specificity compared to our study.^30^ Given the PLR, Tham et al. found a cut‐off value of 320 for PLR with an AUC of 0.90, a sensitivity of 97.66%, and a specificity of 71.43% in patients with head and neck cancer undergoing major surgery.^31^ Compared to our study results, they attained a higher PLR cut‐off with more reliable predictive power in this patient population.

### 4.2 Association between hematological indices and pulmonary embolism severity (s‐PESI score) in COVID‐19 patients

The PESI was developed to estimate 30‐day mortality in patients with acute PTE. The s‐PESI showed similar prognostic accuracy, clinical utility, and more convenience in use compared with the original PESI.^32^ An increased s‐PESI was shown to be associated with a worse PTE prognosis in COVID‐19 patients.^33^ We found that a higher neutrophil count 24 h before PTE diagnosis via CTA is correlated with a higher s‐PESI score. Hence, we postulate that neutrophil count could be a prognostic factor of PTE severity in COVID‐19 patients.

Thoreau et al. study demonstrated that a neutrophil count of more than 7.0 G/L is associated with an increased risk of PTE and also the composite criterion combining a d‐dimer level of more than 2000 ng/mL and neutrophils count of more than 7.0 G/L was associated with an increased risk of death, ICU transfer, and longer hospital stay, nevertheless they found PTE occurrence did not affect time to ICU transfer or death, nor did it influence time to hospital discharge.^34^ Although they observed PTE occurrence does not correlate with worse outcomes, PTE severity as higher neutrophil count could indicate it might have led to a worse prognosis in their patient population which was not assessed in this study.

In another study by Strazzulla et al., the neutrophil count was associated with the diagnosis of acute PTE while no CBC parameters, including neutrophil, were associated with mortality at Day 7.^15^ This disparity between our findings and this study may be attributable to the differing follow‐up duration as the s‐PESI score estimates 30‐day mortality in PTE patients but Strazzulla et al. considered 7‐day mortality in their statistical analysis.

### 4.3 Study limitation

There were some limitations inherent to this study. First, the retrospective design of the study was implicated in the development of some biases and hidden confounders. Selection bias might have occurred as the patient data were collected only after a set of requisites became accessible. Second, we conducted a monocentric study; consequently, our results are not fully applicable to various COVID‐19 patients population. Third, the number of patients included in the study was restricted due to partial loss of information which was inevitable because of the study design. Fourth, the data on the antiviral and anti‐inflammatory medications received by the patients during hospitalization was not available. Therefore, the effect of these drugs on the incidence of PTE and changes in CBC parameters in our COVID‐19 patients could not be assessed as confounders. Finally, any statistics regarding the COVID‐19 vaccination of our subjects were not obtainable; thus, this study could not differentiate the effect of SARS‐CoV‐2 vaccines on the PTE occurrence.

## 5 CONCLUSION

Among hematological indices, NLR and more precisely PLR are cost‐effective and simply calculable markers that can assist physicians in determining whether or not COVID‐19 patients with clinically probable PTE require CTA and the higher neutrophil count can be employed as an indicator of PTE severity in COVID‐19 patients. Further large multicenter and prospective studies are warranted to support these findings and distinguish the effect of SARS‐CoV‐2 variants, antiviral and anti‐inflammatory medications, and COVID‐19 vaccination on the predictive value of CBC parameters for PTE incidence in COVID‐19 patients.

## AUTHOR CONTRIBUTIONS

Hanieh Radkhah, Ensieh Sadat Mansouri, and Milad Gholizadeh Mesgarha were responsible for conceptualization and methodology. Hanieh Radkhah and Milad Gholizadeh Mesgarha supervised the study implementation. Ali Sheikhy was responsible for the formal analysis. Shiva Rahimipour Anaraki was responsible for checking the validity of the study results. Mohamad Mehdi Khadembashiri, Mohamad Amin Khadembashiri, Mohamad Eslami, Tara Mahmoodi, and Behnaz Inanloo had the role of data collection and investigation and wrote the initial draft. Milad Gholizadeh Mesgarha and Shiva Rahimipour Anaraki revised and edited the manuscript and prepared the article's final version.

## CONFLICT OF INTEREST STATEMENT

The authors declare no conflict of interest.

## ETHICS STATEMENT

The study was conducted in accordance with the Declaration of Helsinki and national and institutional standards and has also received ethical approval from the institutional review board of Tehran University of Medical Sciences (Approval number: IR. TUMS. SINAHOSPITAL. REC.1402.005).

## Supporting information

Supporting information.

Supporting information.

## Data Availability

The data that support the findings of this study are available from the corresponding author, M. G. H., upon reasonable request.
