# Supplementary material for: Predictive value of hematological indices on incidence and severity of pulmonary embolism in COVID‐19 patients
Source: Immun Inflamm Dis. 2023 Sep 29;11(9):e1012. doi: 10.1002/iid3.1012 (PMC10540144; doi:10.1002/iid3.1012)
Supplement: Supplementary file 1 — Supporting information. [file IID3-11-e1012-s001.docx]

**Table S1- Different thresholds for NLR**

| **NLR Threshold** | **Sensitivity** | **Specificity** | **PPV** | **NPV** | **YOUNDEN index** |
| --- | --- | --- | --- | --- | --- |
| -1.0000 | 1.000 | 0.000 | 0.356322 | - | 0.000 |
| .5000 | 1.000 | 0.009 | 0.358394 | 1 | 0.009 |
| 1.5000 | .892 | 0.180 | 0.375871 | 0.751118 | 0.072 |
| 2.5000 | .833 | 0.278 | 0.389993 | 0.751118 | 0.112 |
| 3.5000 | .769 | 0.353 | 0.396899 | 0.734086 | 0.122 |
| 4.5000 | .656 | 0.425 | 0.387118 | 0.690597 | 0.081 |
| 5.5000 | .565 | 0.494 | 0.381802 | 0.672049 | 0.059 |
| 6.5000 | .505 | 0.560 | 0.388622 | 0.671569 | 0.065 |
| 7.5000 | .462 | 0.602 | 0.391271 | 0.669097 | 0.064 |
| 8.5000 | .409 | 0.635 | 0.382427 | 0.659727 | 0.043 |
| 9.5000 | .376 | 0.671 | 0.387471 | 0.660164 | 0.047 |
| 10.5000 | .360 | 0.701 | 0.399764 | 0.664222 | 0.061 |
| 11.5000 | .339 | 0.725 | 0.405012 | 0.664346 | 0.063 |
| 12.5000 | .306 | 0.763 | 0.417665 | 0.665393 | 0.070 |
| 13.5000 | .269 | 0.787 | 0.411776 | 0.660488 | 0.056 |
| 14.5000 | .253 | 0.805 | 0.41819 | 0.660653 | 0.058 |
| 15.5000 | .242 | 0.817 | 0.42307 | 0.66076 | 0.059 |
| 16.5000 | .226 | 0.844 | 0.445333 | 0.663306 | 0.070 |
| 17.5000 | .226 | 0.856 | 0.465181 | 0.666445 | 0.082 |
| 18.5000 | .220 | 0.868 | 0.480862 | 0.667992 | 0.089 |
| 19.5000 | .215 | 0.871 | 0.480437 | 0.667231 | 0.086 |
| 20.5000 | .204 | 0.880 | 0.485688 | 0.666487 | 0.085 |
| 21.5000 | .199 | 0.889 | 0.498507 | 0.667246 | 0.088 |
| 22.5000 | .199 | 0.892 | 0.505357 | 0.667992 | 0.091 |
| 23.5000 | .177 | 0.907 | 0.514134 | 0.665803 | 0.085 |
| 24.5000 | .177 | 0.913 | 0.530771 | 0.667266 | 0.091 |
| 25.5000 | .161 | 0.919 | 0.524827 | 0.664399 | 0.080 |
| 26.5000 | .156 | 0.925 | 0.535552 | 0.664423 | 0.081 |
| 27.5000 | .140 | 0.925 | 0.508312 | 0.660189 | 0.065 |
| 28.5000 | .129 | 0.934 | 0.520249 | 0.65957 | 0.063 |
| 29.5000 | .113 | 0.940 | 0.510703 | 0.656879 | 0.053 |
| 31.0000 | .108 | 0.946 | 0.524827 | 0.656948 | 0.054 |
| 32.5000 | .091 | 0.952 | 0.51366 | 0.654329 | 0.043 |
| 33.5000 | .081 | 0.958 | 0.51575 | 0.653085 | 0.039 |
| 34.5000 | .075 | 0.961 | 0.517028 | 0.652471 | 0.036 |
| 35.5000 | .065 | 0.964 | 0.498507 | 0.650552 | 0.029 |
| 36.5000 | .054 | 0.970 | 0.498507 | 0.649361 | 0.024 |
| 37.5000 | .048 | 0.973 | 0.498507 | 0.648772 | 0.021 |
| 39.0000 | .048 | 0.979 | 0.56103 | 0.650169 | 0.027 |
| 42.0000 | .043 | 0.979 | 0.531847 | 0.648886 | 0.022 |
| 44.5000 | .043 | 0.982 | 0.569966 | 0.649582 | 0.025 |
| 45.5000 | .038 | 0.985 | 0.581882 | 0.648999 | 0.023 |
| 47.5000 | .038 | 0.988 | 0.634981 | 0.64969 | 0.026 |
| 50.0000 | .032 | 0.988 | 0.598566 | 0.648421 | 0.020 |
| 53.5000 | .027 | 0.991 | 0.6236 | 0.647848 | 0.018 |
| 67.5000 | .016 | 0.994 | 0.598566 | 0.646027 | 0.010 |
| 93.0000 | .011 | 0.994 | 0.498507 | 0.644779 | 0.005 |
| 122.5000 | .011 | 0.997 | 0.665339 | 0.645468 | 0.008 |
| 191.5000 | .005 | 0.997 | 0.498507 | 0.644227 | 0.002 |
| 246.0000 | 0.000 | 1.000 | - | 0.643678 | 0.000 |

**Table S2- Different threshold for PLR**

| **PLR threshold** | **Sensitivity** | **Specificity** | **PPV** | **NPV** | **YOUNDEN index** |
| --- | --- | --- | --- | --- | --- |
| -1.0000 | 1.000 | 0.000 | 0.356322 | - | 0.000 |
| 1.0000 | .994 | 0.000 | 0.355031 | 0 | -0.006 |
| 3.0000 | .994 | 0.003 | 0.355718 | 0.490503 | -0.003 |
| 5.0000 | .994 | 0.006 | 0.356407 | 0.658171 | 0.000 |
| 7.0000 | .994 | 0.009 | 0.357099 | 0.742809 | 0.003 |
| 12.5000 | .989 | 0.012 | 0.356494 | 0.658171 | 0.001 |
| 18.0000 | .989 | 0.018 | 0.357889 | 0.742809 | 0.007 |
| 22.0000 | .989 | 0.021 | 0.358591 | 0.771142 | 0.010 |
| 25.5000 | .983 | 0.021 | 0.357282 | 0.691961 | 0.004 |
| 26.5000 | .983 | 0.024 | 0.357985 | 0.719672 | 0.007 |
| 27.5000 | .983 | 0.027 | 0.358692 | 0.742809 | 0.010 |
| 29.0000 | .983 | 0.030 | 0.359401 | 0.762418 | 0.013 |
| 30.5000 | .983 | 0.033 | 0.360113 | 0.779248 | 0.016 |
| 32.5000 | .983 | 0.036 | 0.360828 | 0.793852 | 0.019 |
| 36.5000 | .978 | 0.042 | 0.360943 | 0.771142 | 0.019 |
| 40.0000 | .978 | 0.045 | 0.361665 | 0.78309 | 0.022 |
| 41.5000 | .978 | 0.048 | 0.362391 | 0.793852 | 0.025 |
| 42.5000 | .972 | 0.048 | 0.36106 | 0.754944 | 0.020 |
| 43.5000 | .972 | 0.051 | 0.361787 | 0.765986 | 0.023 |
| 45.0000 | .972 | 0.057 | 0.363249 | 0.785331 | 0.029 |
| 47.5000 | .972 | 0.060 | 0.363985 | 0.793852 | 0.032 |
| 50.0000 | .966 | 0.060 | 0.362644 | 0.762418 | 0.026 |
| 51.5000 | .966 | 0.063 | 0.363382 | 0.771142 | 0.029 |
| 55.5000 | .955 | 0.066 | 0.361419 | 0.725838 | 0.021 |
| 60.0000 | .955 | 0.069 | 0.36216 | 0.734594 | 0.024 |
| 61.5000 | .955 | 0.072 | 0.362904 | 0.742809 | 0.027 |
| 62.5000 | .955 | 0.075 | 0.363652 | 0.75053 | 0.030 |
| 64.5000 | .949 | 0.078 | 0.363037 | 0.735533 | 0.027 |
| 67.0000 | .949 | 0.081 | 0.363789 | 0.742809 | 0.030 |
| 68.5000 | .944 | 0.090 | 0.364689 | 0.742809 | 0.034 |
| 71.5000 | .944 | 0.093 | 0.365452 | 0.749023 | 0.037 |
| 76.0000 | .944 | 0.096 | 0.366219 | 0.754944 | 0.040 |
| 78.5000 | .938 | 0.102 | 0.366376 | 0.748471 | 0.040 |
| 81.5000 | .938 | 0.108 | 0.36793 | 0.759078 | 0.046 |
| 84.5000 | .938 | 0.111 | 0.368712 | 0.764053 | 0.049 |
| 86.0000 | .938 | 0.114 | 0.369498 | 0.768827 | 0.052 |
| 87.5000 | .938 | 0.117 | 0.370286 | 0.773411 | 0.055 |
| 88.5000 | .933 | 0.120 | 0.369678 | 0.762418 | 0.052 |
| 89.5000 | .921 | 0.123 | 0.36765 | 0.738178 | 0.044 |
| 90.5000 | .916 | 0.123 | 0.366229 | 0.724627 | 0.038 |
| 92.0000 | .916 | 0.126 | 0.367023 | 0.729409 | 0.041 |
| 93.5000 | .910 | 0.132 | 0.36719 | 0.725838 | 0.042 |
| 94.5000 | .910 | 0.135 | 0.367993 | 0.730287 | 0.045 |
| 95.5000 | .910 | 0.141 | 0.369609 | 0.738766 | 0.051 |
| 96.5000 | .904 | 0.141 | 0.368168 | 0.726898 | 0.045 |
| 98.0000 | .904 | 0.144 | 0.36898 | 0.731058 | 0.048 |
| 99.5000 | .899 | 0.144 | 0.367531 | 0.719672 | 0.043 |
| 100.5000 | .854 | 0.251 | 0.387084 | 0.75671 | 0.105 |
| 101.5000 | .848 | 0.257 | 0.387423 | 0.754085 | 0.106 |
| 102.5000 | .843 | 0.257 | 0.385848 | 0.747278 | 0.100 |
| 104.0000 | .843 | 0.260 | 0.386806 | 0.749456 | 0.103 |
| 106.0000 | .837 | 0.263 | 0.386181 | 0.744986 | 0.101 |
| 108.0000 | .837 | 0.266 | 0.387147 | 0.747127 | 0.104 |
| 109.5000 | .837 | 0.269 | 0.388118 | 0.749232 | 0.107 |
| 111.0000 | .831 | 0.269 | 0.38652 | 0.742809 | 0.101 |
| 112.5000 | .826 | 0.275 | 0.386864 | 0.740738 | 0.101 |
| 113.5000 | .820 | 0.281 | 0.387214 | 0.738766 | 0.102 |
| 114.5000 | .820 | 0.284 | 0.388205 | 0.740803 | 0.105 |
| 116.0000 | .820 | 0.287 | 0.389201 | 0.742809 | 0.108 |
| 118.5000 | .820 | 0.293 | 0.391209 | 0.746728 | 0.114 |
| 120.5000 | .815 | 0.299 | 0.391599 | 0.744724 | 0.114 |
| 122.0000 | .809 | 0.299 | 0.389952 | 0.739008 | 0.108 |
| 124.0000 | .803 | 0.302 | 0.389313 | 0.735319 | 0.106 |
| 125.5000 | .798 | 0.305 | 0.388667 | 0.731738 | 0.103 |
| 127.0000 | .798 | 0.308 | 0.389694 | 0.733649 | 0.106 |
| 128.5000 | .798 | 0.311 | 0.390727 | 0.735533 | 0.109 |
| 129.5000 | .792 | 0.311 | 0.389045 | 0.730169 | 0.104 |
| 130.5000 | .792 | 0.317 | 0.391123 | 0.733905 | 0.110 |
| 132.5000 | .781 | 0.323 | 0.38982 | 0.727223 | 0.104 |
| 135.0000 | .781 | 0.326 | 0.390876 | 0.729047 | 0.107 |
| 136.5000 | .781 | 0.329 | 0.391937 | 0.730847 | 0.110 |
| 137.5000 | .764 | 0.332 | 0.387811 | 0.717859 | 0.096 |
| 138.5000 | .764 | 0.341 | 0.391031 | 0.723229 | 0.105 |
| 139.5000 | .758 | 0.344 | 0.390359 | 0.720257 | 0.103 |
| 141.0000 | .753 | 0.344 | 0.388591 | 0.715602 | 0.097 |
| 142.5000 | .742 | 0.347 | 0.386108 | 0.708262 | 0.089 |
| 143.5000 | .736 | 0.347 | 0.384307 | 0.703798 | 0.083 |
| 144.5000 | .730 | 0.350 | 0.383582 | 0.701192 | 0.081 |
| 146.5000 | .730 | 0.356 | 0.385774 | 0.704731 | 0.087 |
| 149.5000 | .730 | 0.359 | 0.386879 | 0.706469 | 0.090 |
| 152.0000 | .730 | 0.362 | 0.387991 | 0.708187 | 0.093 |
| 153.5000 | .719 | 0.362 | 0.384316 | 0.69968 | 0.081 |
| 156.0000 | .719 | 0.368 | 0.386551 | 0.703113 | 0.087 |
| 158.5000 | .719 | 0.374 | 0.388811 | 0.706469 | 0.093 |
| 159.5000 | .719 | 0.377 | 0.389952 | 0.708119 | 0.096 |
| 161.0000 | .719 | 0.380 | 0.391099 | 0.70975 | 0.099 |
| 162.5000 | .719 | 0.386 | 0.393413 | 0.712959 | 0.105 |
| 163.5000 | .713 | 0.386 | 0.391543 | 0.708889 | 0.100 |
| 164.5000 | .713 | 0.389 | 0.392709 | 0.71048 | 0.103 |
| 165.5000 | .708 | 0.389 | 0.390825 | 0.706469 | 0.097 |
| 166.5000 | .685 | 0.392 | 0.384334 | 0.692504 | 0.078 |
| 167.5000 | .680 | 0.392 | 0.382389 | 0.688722 | 0.072 |
| 168.5000 | .669 | 0.395 | 0.379622 | 0.68293 | 0.064 |
| 169.5000 | .669 | 0.404 | 0.383153 | 0.687776 | 0.073 |
| 170.5000 | .669 | 0.407 | 0.384344 | 0.689359 | 0.076 |
| 171.5000 | .669 | 0.413 | 0.386749 | 0.692477 | 0.082 |
| 172.5000 | .669 | 0.419 | 0.389184 | 0.695532 | 0.088 |
| 173.5000 | .652 | 0.419 | 0.383132 | 0.684929 | 0.071 |
| 174.5000 | .646 | 0.422 | 0.382308 | 0.683008 | 0.068 |
| 175.5000 | .640 | 0.428 | 0.382705 | 0.682648 | 0.069 |
| 176.5000 | .640 | 0.434 | 0.385195 | 0.685649 | 0.075 |
| 178.0000 | .635 | 0.437 | 0.384365 | 0.683786 | 0.072 |
| 179.5000 | .612 | 0.440 | 0.377125 | 0.67224 | 0.052 |
| 180.5000 | .607 | 0.443 | 0.37622 | 0.670561 | 0.050 |
| 182.0000 | .607 | 0.446 | 0.377486 | 0.672047 | 0.053 |
| 183.5000 | .607 | 0.452 | 0.380044 | 0.674979 | 0.059 |
| 184.5000 | .607 | 0.458 | 0.382636 | 0.677859 | 0.065 |
| 186.0000 | .601 | 0.467 | 0.384389 | 0.679001 | 0.068 |
| 187.5000 | .601 | 0.473 | 0.387066 | 0.681771 | 0.074 |
| 188.5000 | .601 | 0.476 | 0.388419 | 0.683138 | 0.077 |
| 189.5000 | .601 | 0.479 | 0.389781 | 0.684494 | 0.080 |
| 190.5000 | .590 | 0.479 | 0.385302 | 0.678464 | 0.069 |
| 192.0000 | .584 | 0.482 | 0.384401 | 0.676853 | 0.066 |
| 194.0000 | .579 | 0.485 | 0.383487 | 0.675269 | 0.064 |
| 196.5000 | .573 | 0.491 | 0.383946 | 0.675055 | 0.064 |
| 198.5000 | .567 | 0.491 | 0.381618 | 0.672181 | 0.058 |
| 200.0000 | .562 | 0.497 | 0.382063 | 0.672009 | 0.059 |
| 202.5000 | .556 | 0.497 | 0.379693 | 0.669195 | 0.053 |
| 204.5000 | .551 | 0.497 | 0.377305 | 0.666405 | 0.048 |
| 205.5000 | .551 | 0.500 | 0.378709 | 0.667738 | 0.051 |
| 207.0000 | .545 | 0.503 | 0.377709 | 0.666305 | 0.048 |
| 208.5000 | .545 | 0.506 | 0.37913 | 0.667624 | 0.051 |
| 209.5000 | .545 | 0.509 | 0.380562 | 0.668931 | 0.054 |
| 210.5000 | .545 | 0.512 | 0.382005 | 0.670229 | 0.057 |
| 212.0000 | .545 | 0.515 | 0.383459 | 0.671517 | 0.060 |
| 214.0000 | .539 | 0.518 | 0.382473 | 0.670087 | 0.057 |
| 215.5000 | .534 | 0.521 | 0.381472 | 0.66868 | 0.055 |
| 216.5000 | .534 | 0.524 | 0.382953 | 0.669949 | 0.058 |
| 217.5000 | .528 | 0.527 | 0.381943 | 0.668559 | 0.055 |
| 218.5000 | .528 | 0.536 | 0.386479 | 0.672293 | 0.064 |
| 219.5000 | .522 | 0.542 | 0.387022 | 0.672134 | 0.064 |
| 220.5000 | .517 | 0.545 | 0.386014 | 0.670769 | 0.062 |
| 221.5000 | .517 | 0.548 | 0.387579 | 0.671978 | 0.065 |
| 222.5000 | .511 | 0.554 | 0.38815 | 0.671826 | 0.065 |
| 224.0000 | .511 | 0.557 | 0.389751 | 0.673013 | 0.068 |
| 225.5000 | .511 | 0.560 | 0.391365 | 0.674192 | 0.071 |
| 226.5000 | .511 | 0.563 | 0.392992 | 0.675363 | 0.074 |
| 227.5000 | .506 | 0.566 | 0.391996 | 0.674019 | 0.071 |
| 228.5000 | .506 | 0.572 | 0.395311 | 0.676327 | 0.077 |
| 229.5000 | .506 | 0.575 | 0.39699 | 0.677469 | 0.080 |
| 232.0000 | .506 | 0.578 | 0.398683 | 0.678603 | 0.083 |
| 234.5000 | .506 | 0.581 | 0.40039 | 0.679729 | 0.086 |
| 235.5000 | .500 | 0.581 | 0.397711 | 0.677265 | 0.081 |
| 236.5000 | .494 | 0.581 | 0.395007 | 0.674817 | 0.075 |
| 238.0000 | .489 | 0.584 | 0.39399 | 0.67352 | 0.073 |
| 239.5000 | .478 | 0.587 | 0.390167 | 0.669853 | 0.064 |
| 241.0000 | .466 | 0.587 | 0.384517 | 0.665131 | 0.053 |
| 242.5000 | .461 | 0.596 | 0.386852 | 0.666181 | 0.056 |
| 243.5000 | .455 | 0.602 | 0.387482 | 0.666101 | 0.057 |
| 244.5000 | .455 | 0.608 | 0.391085 | 0.668299 | 0.063 |
| 245.5000 | .449 | 0.611 | 0.389952 | 0.667114 | 0.060 |
| 246.5000 | .433 | 0.611 | 0.380899 | 0.660384 | 0.043 |
| 247.5000 | .433 | 0.614 | 0.382721 | 0.66148 | 0.046 |
| 249.0000 | .433 | 0.617 | 0.384561 | 0.662569 | 0.049 |
| 250.5000 | .433 | 0.620 | 0.386419 | 0.663651 | 0.052 |
| 251.5000 | .427 | 0.620 | 0.383325 | 0.661448 | 0.047 |
| 253.0000 | .421 | 0.623 | 0.382063 | 0.660341 | 0.044 |
| 254.5000 | .416 | 0.626 | 0.380776 | 0.659249 | 0.041 |
| 256.0000 | .410 | 0.626 | 0.377573 | 0.657096 | 0.036 |
| 257.5000 | .410 | 0.629 | 0.379463 | 0.658171 | 0.039 |
| 258.5000 | .410 | 0.632 | 0.381371 | 0.659239 | 0.042 |
| 259.5000 | .410 | 0.638 | 0.385246 | 0.661355 | 0.048 |
| 260.5000 | .410 | 0.641 | 0.387214 | 0.662403 | 0.051 |
| 262.0000 | .410 | 0.644 | 0.389201 | 0.663445 | 0.054 |
| 264.0000 | .410 | 0.647 | 0.391209 | 0.66448 | 0.057 |
| 265.5000 | .404 | 0.647 | 0.387929 | 0.662364 | 0.051 |
| 266.5000 | .404 | 0.650 | 0.389952 | 0.663396 | 0.054 |
| 267.5000 | .399 | 0.653 | 0.388667 | 0.662325 | 0.052 |
| 269.0000 | .393 | 0.659 | 0.38943 | 0.662287 | 0.052 |
| 272.0000 | .393 | 0.662 | 0.391527 | 0.663301 | 0.055 |
| 276.0000 | .382 | 0.668 | 0.388878 | 0.661212 | 0.050 |
| 279.0000 | .376 | 0.668 | 0.385363 | 0.659181 | 0.044 |
| 280.5000 | .376 | 0.671 | 0.387509 | 0.660186 | 0.047 |
| 282.0000 | .376 | 0.680 | 0.394092 | 0.663164 | 0.056 |
| 283.5000 | .376 | 0.686 | 0.398606 | 0.665121 | 0.062 |
| 284.5000 | .376 | 0.689 | 0.400903 | 0.666091 | 0.065 |
| 285.5000 | .376 | 0.692 | 0.403225 | 0.667055 | 0.068 |
| 286.5000 | .376 | 0.695 | 0.405575 | 0.668014 | 0.071 |
| 287.5000 | .376 | 0.701 | 0.410358 | 0.669914 | 0.077 |
| 288.5000 | .376 | 0.704 | 0.412792 | 0.670857 | 0.080 |
| 290.0000 | .371 | 0.707 | 0.411609 | 0.669813 | 0.077 |
| 291.5000 | .371 | 0.710 | 0.414095 | 0.670748 | 0.080 |
| 292.5000 | .365 | 0.716 | 0.415446 | 0.67064 | 0.081 |
| 293.5000 | .360 | 0.719 | 0.414251 | 0.669616 | 0.078 |
| 295.0000 | .354 | 0.722 | 0.413026 | 0.668603 | 0.075 |
| 297.5000 | .354 | 0.725 | 0.415649 | 0.66952 | 0.078 |
| 300.0000 | .348 | 0.728 | 0.414418 | 0.668515 | 0.076 |
| 301.5000 | .348 | 0.734 | 0.419821 | 0.670329 | 0.082 |
| 302.5000 | .348 | 0.737 | 0.422576 | 0.671229 | 0.085 |
| 303.5000 | .331 | 0.737 | 0.410522 | 0.66557 | 0.068 |
| 305.0000 | .331 | 0.740 | 0.413291 | 0.666472 | 0.071 |
| 306.5000 | .331 | 0.743 | 0.416097 | 0.66737 | 0.074 |
| 307.5000 | .326 | 0.749 | 0.417661 | 0.667295 | 0.074 |
| 309.0000 | .326 | 0.751 | 0.420577 | 0.668181 | 0.077 |
| 310.5000 | .326 | 0.754 | 0.423534 | 0.669062 | 0.080 |
| 312.5000 | .326 | 0.760 | 0.429574 | 0.67081 | 0.086 |
| 315.5000 | .320 | 0.760 | 0.425317 | 0.668975 | 0.081 |
| 317.5000 | .320 | 0.763 | 0.428395 | 0.669844 | 0.084 |
| 319.5000 | .315 | 0.763 | 0.424066 | 0.668021 | 0.078 |
| 322.5000 | .309 | 0.766 | 0.422777 | 0.667078 | 0.075 |
| 324.5000 | .309 | 0.769 | 0.425929 | 0.667944 | 0.078 |
| 326.0000 | .303 | 0.769 | 0.421449 | 0.666145 | 0.073 |
| 327.5000 | .303 | 0.772 | 0.424639 | 0.667008 | 0.076 |
| 328.5000 | .298 | 0.772 | 0.420079 | 0.665222 | 0.070 |
| 332.0000 | .298 | 0.775 | 0.423309 | 0.666083 | 0.073 |
| 339.5000 | .292 | 0.778 | 0.421937 | 0.665167 | 0.071 |
| 346.0000 | .287 | 0.781 | 0.42052 | 0.664261 | 0.068 |
| 348.5000 | .287 | 0.784 | 0.423884 | 0.665113 | 0.071 |
| 349.5000 | .287 | 0.787 | 0.427304 | 0.665961 | 0.074 |
| 351.0000 | .287 | 0.790 | 0.430778 | 0.666805 | 0.077 |
| 352.5000 | .281 | 0.790 | 0.425929 | 0.66506 | 0.071 |
| 355.0000 | .281 | 0.793 | 0.429451 | 0.665902 | 0.074 |
| 357.5000 | .275 | 0.793 | 0.424508 | 0.664168 | 0.069 |
| 359.5000 | .275 | 0.796 | 0.428079 | 0.665008 | 0.072 |
| 362.0000 | .264 | 0.799 | 0.421516 | 0.662411 | 0.063 |
| 363.5000 | .258 | 0.799 | 0.416281 | 0.660709 | 0.058 |
| 364.5000 | .258 | 0.802 | 0.41994 | 0.661546 | 0.061 |
| 366.0000 | .258 | 0.808 | 0.427453 | 0.663209 | 0.067 |
| 368.0000 | .253 | 0.811 | 0.425929 | 0.662348 | 0.064 |
| 370.0000 | .253 | 0.817 | 0.433836 | 0.663991 | 0.070 |
| 374.5000 | .247 | 0.817 | 0.428324 | 0.662318 | 0.065 |
| 378.5000 | .247 | 0.820 | 0.432376 | 0.663135 | 0.068 |
| 383.5000 | .247 | 0.823 | 0.436506 | 0.663948 | 0.071 |
| 388.5000 | .242 | 0.826 | 0.435056 | 0.663099 | 0.068 |
| 390.5000 | .242 | 0.829 | 0.439336 | 0.663906 | 0.071 |
| 397.5000 | .242 | 0.832 | 0.4437 | 0.66471 | 0.074 |
| 403.5000 | .236 | 0.832 | 0.4379 | 0.663063 | 0.068 |
| 405.0000 | .230 | 0.832 | 0.431978 | 0.661424 | 0.063 |
| 406.5000 | .230 | 0.835 | 0.436404 | 0.662228 | 0.066 |
| 407.5000 | .225 | 0.835 | 0.430341 | 0.660599 | 0.060 |
| 408.5000 | .219 | 0.835 | 0.424145 | 0.658978 | 0.054 |
| 410.0000 | .213 | 0.835 | 0.417814 | 0.657366 | 0.049 |
| 412.0000 | .213 | 0.838 | 0.422284 | 0.658171 | 0.052 |
| 413.5000 | .208 | 0.838 | 0.415792 | 0.656568 | 0.046 |
| 416.5000 | .208 | 0.841 | 0.420339 | 0.657371 | 0.049 |
| 425.5000 | .208 | 0.844 | 0.424987 | 0.658171 | 0.052 |
| 433.5000 | .202 | 0.844 | 0.418306 | 0.656579 | 0.047 |
| 436.0000 | .202 | 0.847 | 0.423038 | 0.657377 | 0.050 |
| 438.0000 | .197 | 0.847 | 0.416178 | 0.655795 | 0.044 |
| 439.5000 | .191 | 0.850 | 0.413948 | 0.655017 | 0.041 |
| 441.0000 | .185 | 0.850 | 0.406725 | 0.653452 | 0.036 |
| 442.5000 | .185 | 0.853 | 0.411609 | 0.654247 | 0.039 |
| 444.0000 | .185 | 0.856 | 0.416611 | 0.655039 | 0.042 |
| 449.0000 | .185 | 0.859 | 0.421737 | 0.655828 | 0.045 |
| 455.0000 | .180 | 0.859 | 0.414251 | 0.654275 | 0.039 |
| 458.5000 | .174 | 0.859 | 0.406569 | 0.652729 | 0.033 |
| 460.5000 | .169 | 0.859 | 0.398683 | 0.651191 | 0.028 |
| 463.5000 | .169 | 0.862 | 0.403849 | 0.65198 | 0.031 |
| 466.5000 | .169 | 0.871 | 0.420186 | 0.654328 | 0.040 |
| 467.5000 | .169 | 0.874 | 0.425929 | 0.655103 | 0.043 |
| 468.5000 | .163 | 0.874 | 0.417661 | 0.65358 | 0.037 |
| 472.0000 | .163 | 0.877 | 0.423534 | 0.654354 | 0.040 |
| 479.5000 | .163 | 0.880 | 0.429574 | 0.655124 | 0.043 |
| 485.0000 | .163 | 0.883 | 0.435788 | 0.655891 | 0.046 |
| 487.5000 | .163 | 0.886 | 0.442185 | 0.656654 | 0.049 |
| 489.5000 | .157 | 0.889 | 0.440109 | 0.655906 | 0.047 |
| 492.0000 | .152 | 0.889 | 0.431168 | 0.654405 | 0.041 |
| 498.0000 | .152 | 0.892 | 0.4379 | 0.655165 | 0.044 |
| 508.5000 | .152 | 0.895 | 0.444846 | 0.655921 | 0.047 |
| 520.0000 | .146 | 0.898 | 0.442685 | 0.655185 | 0.044 |
| 529.0000 | .146 | 0.901 | 0.450063 | 0.655936 | 0.047 |
| 536.5000 | .146 | 0.904 | 0.45769 | 0.656684 | 0.050 |
| 541.0000 | .140 | 0.904 | 0.447972 | 0.655205 | 0.045 |
| 542.5000 | .140 | 0.907 | 0.455836 | 0.655951 | 0.048 |
| 543.5000 | .135 | 0.910 | 0.453844 | 0.655224 | 0.045 |
| 546.5000 | .135 | 0.913 | 0.46226 | 0.655965 | 0.048 |
| 550.5000 | .135 | 0.916 | 0.470993 | 0.656704 | 0.051 |
| 554.0000 | .129 | 0.919 | 0.469451 | 0.65598 | 0.048 |
| 556.5000 | .118 | 0.919 | 0.446871 | 0.653081 | 0.037 |
| 557.5000 | .118 | 0.922 | 0.456217 | 0.653817 | 0.040 |
| 558.5000 | .118 | 0.925 | 0.465963 | 0.654551 | 0.043 |
| 561.0000 | .112 | 0.925 | 0.453844 | 0.653113 | 0.038 |
| 564.0000 | .101 | 0.925 | 0.427879 | 0.650258 | 0.026 |
| 566.5000 | .101 | 0.928 | 0.4379 | 0.650993 | 0.029 |
| 568.5000 | .101 | 0.931 | 0.448402 | 0.651724 | 0.032 |
| 571.0000 | .101 | 0.934 | 0.45942 | 0.652452 | 0.035 |
| 582.5000 | .090 | 0.940 | 0.453844 | 0.651083 | 0.030 |
| 596.5000 | .084 | 0.940 | 0.4379 | 0.649684 | 0.024 |
| 606.5000 | .079 | 0.940 | 0.420997 | 0.648291 | 0.019 |
| 629.5000 | .073 | 0.943 | 0.415446 | 0.647629 | 0.016 |
| 653.0000 | .067 | 0.943 | 0.396148 | 0.646249 | 0.011 |
| 666.0000 | .067 | 0.946 | 0.409152 | 0.646973 | 0.014 |
| 688.5000 | .067 | 0.949 | 0.423038 | 0.647695 | 0.017 |
| 704.5000 | .056 | 0.949 | 0.379273 | 0.644957 | 0.005 |
| 707.5000 | .051 | 0.952 | 0.368799 | 0.644319 | 0.003 |
| 710.5000 | .045 | 0.952 | 0.341829 | 0.642966 | -0.003 |
| 712.0000 | .039 | 0.952 | 0.312451 | 0.641618 | -0.009 |
| 713.5000 | .039 | 0.955 | 0.32648 | 0.64234 | -0.006 |
| 716.5000 | .039 | 0.958 | 0.341829 | 0.643059 | -0.003 |
| 728.5000 | .039 | 0.961 | 0.358692 | 0.643775 | 0.000 |
| 749.5000 | .034 | 0.961 | 0.324055 | 0.642436 | -0.005 |
| 761.5000 | .028 | 0.961 | 0.285464 | 0.641103 | -0.011 |
| 789.0000 | .028 | 0.964 | 0.302067 | 0.641819 | -0.008 |
| 834.5000 | .022 | 0.964 | 0.257191 | 0.640493 | -0.013 |
| 872.5000 | .022 | 0.967 | 0.274162 | 0.641206 | -0.010 |
| 905.5000 | .017 | 0.967 | 0.220752 | 0.639887 | -0.016 |
| 922.0000 | .017 | 0.970 | 0.237582 | 0.640599 | -0.013 |
| 934.5000 | .011 | 0.973 | 0.187538 | 0.639996 | -0.016 |
| 1013.0000 | .011 | 0.976 | 0.206148 | 0.640704 | -0.013 |
| 1083.0000 | .011 | 0.982 | 0.257191 | 0.642111 | -0.007 |
| 1164.5000 | .011 | 0.985 | 0.293531 | 0.64281 | -0.004 |
| 1256.5000 | .006 | 0.985 | 0.17201 | 0.641508 | -0.009 |
| 1289.0000 | .006 | 0.991 | 0.257191 | 0.642901 | -0.003 |
| 1339.0000 | .006 | 0.994 | 0.341829 | 0.643593 | 0.000 |
| 1368.5000 | .006 | 0.997 | 0.509497 | 0.644282 | 0.003 |
| 1823.5000 | .006 | 1.000 | 1 | 0.644969 | 0.006 |
| 2279.0000 | 0.000 | 1.000 | - | 0.643678 | 0.000 |
