# Supplementary material for: Predictive value of hematological indices on incidence and severity of pulmonary embolism in COVID‐19 patients
Source: Immun Inflamm Dis. 2023 Sep 29;11(9):e1012. doi: 10.1002/iid3.1012 (PMC10540144; doi:10.1002/iid3.1012)
Supplement: Supplementary file 2 — Supporting information. [file IID3-11-e1012-s002.docx]

STROBE Statement—checklist of items that should be included in reports of observational studies

|  | Item No. | Recommendation | Page  No. | Relevant text from manuscript |
| --- | --- | --- | --- | --- |
| **Title and abstract** | 1 | (*a*) Indicate the study’s design with a commonly used term in the title or the abstract | 3 | retrospective cohort study |
|  |  | (*b*) Provide in the abstract an informative and balanced summary of what was done and what was found | 3 | We discovered that among individuals with a probable PTE, males and those with higher platelet-to-lymphocyte (PLR) and neutrophil-to-lymphocyte (NLR) ratios had a greater likelihood of PTE incidence. PLR was a significant and independent predictor of PTE. Moreover, a higher neutrophil count was associated with a higher s-PESI score in COVID-19 patients developing PTE. |
| Introduction | | | |  |
| Background/rationale | 2 | Explain the scientific background and rationale for the investigation being reported | 4 | The severe acute respiratory syndrome coronavirus 2 (SARS-CoV-2) outbreak that began in Wuhan, China, in December 2019 rapidly spread worldwide, insofar as the World Health Organization has declared COVID-19 to be a pandemic and a public health emergency of international concern (1-4). Although the disease is typically associated with respiratory tract involvement, the virus also can affect other systems including hematological and cardiovascular systems through different hypothesized mechanisms (5, 6). One of the most important underlying mechanisms is an increased level of inflammatory mediators (7). This inflammatory process is accompanied by thrombotic events, including pulmonary thromboembolism (PTE), in COVID-19 patients (8-10).  Pulmonary thromboembolism is a cause of mortality among COVID-19 complications. While the case fatality rate for COVID-19 ranges from 2% to 3%, the mortality rate for COVID-19 patients who develop PTE is 45.1% (11). This remarkable complication is reported in 15.3 percent of COVID-19 cases (12). Due to the high mortality and incidence of PTE, as well as the fact that prompt treatment is highly effective and has been shown to significantly impact clinical outcomes, early diagnosis of PTE is crucial (12, 13).  Nevertheless, PTE diagnosis in COVID-19 is challenging for a variety of reasons. First, PTE and COVID-19 clinical manifestations may overlap (14). Second, hospital overcrowding during the resurgence of the COVID-19 epidemic, costs, and lack of availability in all centers make chest CT angiography inaccessible for all patients with probable PTE (15). Third, available clinical prediction models for PTE do not apply to COVID-19 patients. Therefore, their use as the sole diagnostic screening tool in clinical practice is not recommended. New clinical probability models for PTE that have been validated in COVID-19 patients are consequently required (16).  As previously stated, thrombotic events accompany the inflammatory process in patients with COVID-19 (8-10). Notably, hematological ratios are one of the valuable, inexpensive, and widely-examined markers of inflammation (17). In addition, circulating biomarkers of inflammation, such as Neutrophil-to-Lymphocyte ratio (NLR) and Platelet-to-Lymphocyte ratio (PLR), have been proposed as reliable prognosticators for both COVID-19 and PTE patients (18-20). Despite the importance of the aforementioned hematologic markers, there are very few studies focusing on the prediction of PTE occurrence or PTE severity in COVID-19 patients with probable PTE |
| Objectives | 3 | State specific objectives, including any prespecified hypotheses | 5 | we conducted an exploratory analysis to evaluate the potential role of these parameters as predictors of thromboembolism in COVID-19 patients and the correlation of CBC parameters with the simplified-PESI score as an indicator of PTE severity. |
| Methods | | | |  |
| Study design | 4 | Present key elements of study design early in the paper | 5 | On the routine basis in the hospital, COVID-19 patients with probable PTE due to changes in their clinical condition who were patients with poor response to treatment, hemoptysis, and spontaneous deterioration, comprising sudden respiratory distress, tachycardia, sudden drop in blood pressure, or O2 saturation, underwent Chest CT angiography (CTA) immediately after diagnostic doubt (21). All these patients received prophylactic anticoagulant and every patient who was diagnosed with PTE was treated by the standard protocol of PTE treatment. Among these above-mentioned patients, cases with a confirmed diagnosis of SARS-CoV-2 infection based on positive chest CT scan findings or positive nasopharyngeal reverse transcriptase-polymerase chain reaction (RT-PCR) test at the general ward & ICU of Sina hospital, Tehran, Iran, between “March- 2019” and “July- 2022”, were enrolled in this observational, analytical, retrospective cohort study and based on the results of CTA, they were divided into the PTE and non-PTE groups.  Subsequently, patients meeting exclusion criteria consist of 1. Previous history of deep vein thrombosis (DVT) or PTE, 2. Severe sepsis, 3. End-stage renal disease, 4. Cirrhosis, 5. Hemoglobinopathies (e.g. Thalassemia), 6. Platelet disorders (e.g. Immune thrombocytopenia, Thrombotic thrombocytopenic purpura), 7. Haematological malignancies (e.g. ALL, CLL, lymphoma), 8. Immunosuppressive drugs use, and 9. Blood products transfusion within 48 hours of blood sampling were excluded, and the remaining patients were included in the final sample. |
| Setting | 5 | Describe the setting, locations, and relevant dates, including periods of recruitment, exposure, follow-up, and data collection | 5,6 | COVID-19 patients data were collected during two years of the pandemic in Iran which encompassed six peaks of disease and various subtypes of SARS-CoV-2 infections. Patients’ blood samples were checked for complete blood count (CBC) (including white blood cell (WBC) count and differentials (Neutrophil and Lymphocyte), platelets count , Hemoglobin (Hb), red cell distribution width (RDW), mean platelet volume (MPV)), D-dimer, C-reactive protein (CRP), and ferritin, then NLR and PLR calculated and analysis was performed on the laboratory results of 24 hours prior to CTA. Moreover, the simplified pulmonary embolism severity index score (simplified PESI score) was estimated for the patients with confirmed PTE on the day of its diagnosis. |
| Participants | 6 | (*a*) *Cohort study*—Give the eligibility criteria, and the sources and methods of selection of participants. Describe methods of follow-up  *Case-control study*—Give the eligibility criteria, and the sources and methods of case ascertainment and control selection. Give the rationale for the choice of cases and controls  *Cross-sectional study*—Give the eligibility criteria, and the sources and methods of selection of participants | 5 | On the routine basis in the hospital, COVID-19 patients with probable PTE due to changes in their clinical condition who were patients with poor response to treatment, hemoptysis, and spontaneous deterioration, comprising sudden respiratory distress, tachycardia, sudden drop in blood pressure, or O2 saturation, underwent Chest CT angiography (CTA) immediately after diagnostic doubt (21). All these patients received prophylactic anticoagulant and every patient who was diagnosed with PTE was treated by the standard protocol of PTE treatment. Among these above-mentioned patients, cases with a confirmed diagnosis of SARS-CoV-2 infection based on positive chest CT scan findings or positive nasopharyngeal reverse transcriptase-polymerase chain reaction (RT-PCR) test at the general ward & ICU of Sina hospital, Tehran, Iran, between “March- 2019” and “July- 2022”, were enrolled in this observational, analytical, retrospective cohort study and based on the results of CTA, they were divided into the PTE and non-PTE groups.  Subsequently, patients meeting exclusion criteria consist of 1. Previous history of deep vein thrombosis (DVT) or PTE, 2. Severe sepsis, 3. End-stage renal disease, 4. Cirrhosis, 5. Hemoglobinopathies (e.g. Thalassemia), 6. Platelet disorders (e.g. Immune thrombocytopenia, Thrombotic thrombocytopenic purpura), 7. Haematological malignancies (e.g. ALL, CLL, lymphoma), 8. Immunosuppressive drugs use, and 9. Blood products transfusion within 48 hours of blood sampling were excluded, and the remaining patients were included in the final sample. |
|  |  | (*b*) *Cohort study*—For matched studies, give matching criteria and number of exposed and unexposed  *Case-control study*—For matched studies, give matching criteria and the number of controls per case | 5 | On the routine basis in the hospital, COVID-19 patients with probable PTE due to changes in their clinical condition who were patients with poor response to treatment, hemoptysis, and spontaneous deterioration, comprising sudden respiratory distress, tachycardia, sudden drop in blood pressure, or O2 saturation, underwent Chest CT angiography (CTA) immediately after diagnostic doubt (21). All these patients received prophylactic anticoagulant and every patient who was diagnosed with PTE was treated by the standard protocol of PTE treatment. Among these above-mentioned patients, cases with a confirmed diagnosis of SARS-CoV-2 infection based on positive chest CT scan findings or positive nasopharyngeal reverse transcriptase-polymerase chain reaction (RT-PCR) test at the general ward & ICU of Sina hospital, Tehran, Iran, between “March- 2019” and “July- 2022”, were enrolled in this observational, analytical, retrospective cohort study and based on the results of CTA, they were divided into the PTE and non-PTE groups.  Subsequently, patients meeting exclusion criteria consist of 1. Previous history of deep vein thrombosis (DVT) or PTE, 2. Severe sepsis, 3. End-stage renal disease, 4. Cirrhosis, 5. Hemoglobinopathies (e.g. Thalassemia), 6. Platelet disorders (e.g. Immune thrombocytopenia, Thrombotic thrombocytopenic purpura), 7. Haematological malignancies (e.g. ALL, CLL, lymphoma), 8. Immunosuppressive drugs use, and 9. Blood products transfusion within 48 hours of blood sampling were excluded, and the remaining patients were included in the final sample. |
| Variables | 7 | Clearly define all outcomes, exposures, predictors, potential confounders, and effect modifiers. Give diagnostic criteria, if applicable | 5,6 | 2.1. Participants and Study Design  On the routine basis in the hospital, COVID-19 patients with probable PTE due to changes in their clinical condition who were patients with poor response to treatment, hemoptysis, and spontaneous deterioration, comprising sudden respiratory distress, tachycardia, sudden drop in blood pressure, or O2 saturation, underwent Chest CT angiography (CTA) immediately after diagnostic doubt (21). All these patients received prophylactic anticoagulant and every patient who was diagnosed with PTE was treated by the standard protocol of PTE treatment. Among these above-mentioned patients, cases with a confirmed diagnosis of SARS-CoV-2 infection based on positive chest CT scan findings or positive nasopharyngeal reverse transcriptase-polymerase chain reaction (RT-PCR) test at the general ward & ICU of Sina hospital, Tehran, Iran, between “March- 2019” and “July- 2022”, were enrolled in this observational, analytical, retrospective cohort study and based on the results of CTA, they were divided into the PTE and non-PTE groups.  Subsequently, patients meeting exclusion criteria consist of 1. Previous history of deep vein thrombosis (DVT) or PTE, 2. Severe sepsis, 3. End-stage renal disease, 4. Cirrhosis, 5. Hemoglobinopathies (e.g. Thalassemia), 6. Platelet disorders (e.g. Immune thrombocytopenia, Thrombotic thrombocytopenic purpura), 7. Haematological malignancies (e.g. ALL, CLL, lymphoma), 8. Immunosuppressive drugs use, and 9. Blood products transfusion within 48 hours of blood sampling were excluded, and the remaining patients were included in the final sample.  2.2. Data Collection  COVID-19 patients data were collected during two years of the pandemic in Iran which encompassed six peaks of disease and various subtypes of SARS-CoV-2 infections. Patients’ blood samples were checked for complete blood count (CBC) (including white blood cell (WBC) count and differentials (Neutrophil and Lymphocyte), platelets count , Hemoglobin (Hb), red cell distribution width (RDW), mean platelet volume (MPV)), D-dimer, C-reactive protein (CRP), and ferritin, then NLR and PLR calculated and analysis was performed on the laboratory results of 24 hours prior to CTA. Moreover, the simplified pulmonary embolism severity index score (simplified PESI score) was estimated for the patients with confirmed PTE on the day of its diagnosis. |
| Data sources/ measurement | 8* | For each variable of interest, give sources of data and details of methods of assessment (measurement). Describe comparability of assessment methods if there is more than one group | 6 | COVID-19 patients data were collected during two years of the pandemic in Iran which encompassed six peaks of disease and various subtypes of SARS-CoV-2 infections. Patients’ blood samples were checked for complete blood count (CBC) (including white blood cell (WBC) count and differentials (Neutrophil and Lymphocyte), platelets count , Hemoglobin (Hb), red cell distribution width (RDW), mean platelet volume (MPV)), D-dimer, C-reactive protein (CRP), and ferritin, then NLR and PLR calculated and analysis was performed on the laboratory results of 24 hours prior to CTA. Moreover, the simplified pulmonary embolism severity index score (simplified PESI score) was estimated for the patients with confirmed PTE on the day of its diagnosis.  Statistical Analysis  Statistical analysis was done on the aforementioned laboratory data, simplified PESI score in addition to patients’ age, and gender in each PTE and non-PTE group. All the collected data were entered into IBM SPSS Statistics for Windows, version 22.0 (Armonk, NY: IBM Corp.)) and R version 4.0.3 (Vienna, Austria) for analysis.  Qualitative data were described as absolute frequencies and percentages, and quantitative data were reported as mean and standard deviation or median and interquartile range according to their distribution. The normality of the variables was assessed using histogram charts as well as central tendency and dispersion measures. The qualitative variables were compared between the ‘non-PTE’ and ‘PTE’ groups applying the chi-square test, and the quantitative variables were compared between the two above-mentioned groups using T-test for normally distributed and Mann–Whitney U test for skewed distributed variables.  The univariate effect of covariates on PTE was assessed by the “Logistic regression” model and reported as an Odds ratio (OR) with 95% confidence interval (95% CI). Covariates with P values less than 0.1 in the univariate Logistic regression analyses were entered into multivariate Logistic regression analysis. The backward elimination method was considered for multivariate Logistic regression analysis to locate predictors of PTE.  Receiver operator characteristic (ROC) curves were generated to estimate the accuracy of each covariate in predicting PTE. The area under each ROC curve (AUC) was calculated to identify an accurate prognostic covariate. |
| Bias | 9 | Describe any efforts to address potential sources of bias | 5 | patients meeting exclusion criteria consist of 1. Previous history of deep vein thrombosis (DVT) or PTE, 2. Severe sepsis, 3. End-stage renal disease, 4. Cirrhosis, 5. Hemoglobinopathies (e.g. Thalassemia), 6. Platelet disorders (e.g. Immune thrombocytopenia, Thrombotic thrombocytopenic purpura), 7. Haematological malignancies (e.g. ALL, CLL, lymphoma), 8. Immunosuppressive drugs use, and 9. Blood products transfusion within 48 hours of blood sampling were excluded, and the remaining patients were included in the final sample. |
| Study size | 10 | Explain how the study size was arrived at | 5 | On the routine basis in the hospital, COVID-19 patients with probable PTE due to changes in their clinical condition who were patients with poor response to treatment, hemoptysis, and spontaneous deterioration, comprising sudden respiratory distress, tachycardia, sudden drop in blood pressure, or O2 saturation, underwent Chest CT angiography (CTA) immediately after diagnostic doubt (21). All these patients received prophylactic anticoagulant and every patient who was diagnosed with PTE was treated by the standard protocol of PTE treatment. Among these above-mentioned patients, cases with a confirmed diagnosis of SARS-CoV-2 infection based on positive chest CT scan findings or positive nasopharyngeal reverse transcriptase-polymerase chain reaction (RT-PCR) test at the general ward & ICU of Sina hospital, Tehran, Iran, between “March- 2019” and “July- 2022”, were enrolled in this observational, analytical, retrospective cohort study and based on the results of CTA, they were divided into the PTE and non-PTE groups. |

Continued on next page

| Quantitative variables | 11 | Explain how quantitative variables were handled in the analyses. If applicable, describe which groupings were chosen and why | 6 | Statistical analysis was done on the aforementioned laboratory data, simplified PESI score in addition to patients’ age, and gender in each PTE and non-PTE group. All the collected data were entered into IBM SPSS Statistics for Windows, version 22.0 (Armonk, NY: IBM Corp.)) and R version 4.0.3 (Vienna, Austria) for analysis.  Qualitative data were described as absolute frequencies and percentages, and quantitative data were reported as mean and standard deviation or median and interquartile range according to their distribution. The normality of the variables was assessed using histogram charts as well as central tendency and dispersion measures. The qualitative variables were compared between the ‘non-PTE’ and ‘PTE’ groups applying the chi-square test, and the quantitative variables were compared between the two above-mentioned groups using T-test for normally distributed and Mann–Whitney U test for skewed distributed variables.  The univariate effect of covariates on PTE was assessed by the “Logistic regression” model and reported as an Odds ratio (OR) with 95% confidence interval (95% CI). Covariates with P values less than 0.1 in the univariate Logistic regression analyses were entered into multivariate Logistic regression analysis. The backward elimination method was considered for multivariate Logistic regression analysis to locate predictors of PTE.  Receiver operator characteristic (ROC) curves were generated to estimate the accuracy of each covariate in predicting PTE. The area under each ROC curve (AUC) was calculated to identify an accurate prognostic covariate. |
| --- | --- | --- | --- | --- |
| Statistical methods | 12 | (*a*) Describe all statistical methods, including those used to control for confounding | 6 | Statistical analysis was done on the aforementioned laboratory data, simplified PESI score in addition to patients’ age, and gender in each PTE and non-PTE group. All the collected data were entered into IBM SPSS Statistics for Windows, version 22.0 (Armonk, NY: IBM Corp.)) and R version 4.0.3 (Vienna, Austria) for analysis.  Qualitative data were described as absolute frequencies and percentages, and quantitative data were reported as mean and standard deviation or median and interquartile range according to their distribution. The normality of the variables was assessed using histogram charts as well as central tendency and dispersion measures. The qualitative variables were compared between the ‘non-PTE’ and ‘PTE’ groups applying the chi-square test, and the quantitative variables were compared between the two above-mentioned groups using T-test for normally distributed and Mann–Whitney U test for skewed distributed variables.  The univariate effect of covariates on PTE was assessed by the “Logistic regression” model and reported as an Odds ratio (OR) with 95% confidence interval (95% CI). Covariates with P values less than 0.1 in the univariate Logistic regression analyses were entered into multivariate Logistic regression analysis. The backward elimination method was considered for multivariate Logistic regression analysis to locate predictors of PTE.  Receiver operator characteristic (ROC) curves were generated to estimate the accuracy of each covariate in predicting PTE. The area under each ROC curve (AUC) was calculated to identify an accurate prognostic covariate. |
|  |  | (*b*) Describe any methods used to examine subgroups and interactions | 6 | Statistical analysis was done on the aforementioned laboratory data, simplified PESI score in addition to patients’ age, and gender in each PTE and non-PTE group. All the collected data were entered into IBM SPSS Statistics for Windows, version 22.0 (Armonk, NY: IBM Corp.)) and R version 4.0.3 (Vienna, Austria) for analysis.  Qualitative data were described as absolute frequencies and percentages, and quantitative data were reported as mean and standard deviation or median and interquartile range according to their distribution. The normality of the variables was assessed using histogram charts as well as central tendency and dispersion measures. The qualitative variables were compared between the ‘non-PTE’ and ‘PTE’ groups applying the chi-square test, and the quantitative variables were compared between the two above-mentioned groups using T-test for normally distributed and Mann–Whitney U test for skewed distributed variables.  The univariate effect of covariates on PTE was assessed by the “Logistic regression” model and reported as an Odds ratio (OR) with 95% confidence interval (95% CI). Covariates with P values less than 0.1 in the univariate Logistic regression analyses were entered into multivariate Logistic regression analysis. The backward elimination method was considered for multivariate Logistic regression analysis to locate predictors of PTE.  Receiver operator characteristic (ROC) curves were generated to estimate the accuracy of each covariate in predicting PTE. The area under each ROC curve (AUC) was calculated to identify an accurate prognostic covariate. |
|  |  | (*c*) Explain how missing data were addressed | 6 | Statistical analysis was done on the aforementioned laboratory data, simplified PESI score in addition to patients’ age, and gender in each PTE and non-PTE group. All the collected data were entered into IBM SPSS Statistics for Windows, version 22.0 (Armonk, NY: IBM Corp.)) and R version 4.0.3 (Vienna, Austria) for analysis.  Qualitative data were described as absolute frequencies and percentages, and quantitative data were reported as mean and standard deviation or median and interquartile range according to their distribution. The normality of the variables was assessed using histogram charts as well as central tendency and dispersion measures. The qualitative variables were compared between the ‘non-PTE’ and ‘PTE’ groups applying the chi-square test, and the quantitative variables were compared between the two above-mentioned groups using T-test for normally distributed and Mann–Whitney U test for skewed distributed variables.  The univariate effect of covariates on PTE was assessed by the “Logistic regression” model and reported as an Odds ratio (OR) with 95% confidence interval (95% CI). Covariates with P values less than 0.1 in the univariate Logistic regression analyses were entered into multivariate Logistic regression analysis. The backward elimination method was considered for multivariate Logistic regression analysis to locate predictors of PTE.  Receiver operator characteristic (ROC) curves were generated to estimate the accuracy of each covariate in predicting PTE. The area under each ROC curve (AUC) was calculated to identify an accurate prognostic covariate. |
|  |  | (*d*) *Cohort study*—If applicable, explain how loss to follow-up was addressed  *Case-control study*—If applicable, explain how matching of cases and controls was addressed  *Cross-sectional study*—If applicable, describe analytical methods taking account of sampling strategy | 6 |  |
|  |  | (*e*) Describe any sensitivity analyses | 6 | ROC curve analysis |
| Results | | | | |
| Participants | 13* | (a) Report numbers of individuals at each stage of study—eg numbers potentially eligible, examined for eligibility, confirmed eligible, included in the study, completing follow-up, and analysed | 7,16 | Among 464 COVID-19 patients who underwent CTA, 28 patients were excluded from the study according to inclusion and exclusion criteria. Of these excluded patients, nine patients were diagnosed with PTE. The demographic and characteristics of included patients are shown in Table 1. |
|  |  | (b) Give reasons for non-participation at each stage | 7 | 28 patients were excluded from the study according to inclusion and exclusion criteria. Of these excluded patients, nine patients were diagnosed with PTE. |
|  |  | (c) Consider use of a flow diagram | - |  |
| Descriptive data | 14* | (a) Give characteristics of study participants (eg demographic, clinical, social) and information on exposures and potential confounders | 16 | Table 1 |
|  |  | (b) Indicate number of participants with missing data for each variable of interest | - |  |
|  |  | (c) *Cohort study*—Summarise follow-up time (eg, average and total amount) | - |  |
| Outcome data | 15* | *Cohort study*—Report numbers of outcome events or summary measures over time | 7,8 | In brief, male gender were significantly correlated with PTE occurrence in patients with underlying COVID-19 (81.7% vs. 41.6%, P-Value<0.001). The median age had no significant differences between two groups. Except for NLR and PLR, other lab data including MPV had no significant differences between two studied groups. NLR was significantly higher in PTE group (235.50 vs. 205.50, P-Value: 0.027). Comparably, PLR was significantly higher in COVID patients which led to PTE (13.65 vs. 9.94, P-Value:0.037).  3.2. Stepwise logistic regression analyses of the disease determinants  All variables with a p-value ≤0.1 in univariate analyses (Table 2) were included in stepwise logistic regression (Table 3). According to the univariate analysis, patients with higher Neutrophil percentage, PLR, and NLR 24 hours prior to CTA had a higher risk of PTE incidence; however, they were not statistically significant except PLR. The OR was estimated as 1.01 (0.99-1.03) for Neutrophil percentage, 1.01 (1.00-1,02) for PLR, and 1.01 (1.00-1.02) for NLR. In contrast, patients with higher lymphocyte percentage had a lower chance of PTE although it was not statistically significant. The OR for mentioned protective factor was estimated as 0.98 (0.96-1.00). In the multivariate analysis, PLR and NLR 24 hours prior to CTA were revealed as potential determinants of PTE (OR:1.001 (1.000-1.002) and OR:1.006 (0.998- 1.018), respectively).  3.3. Receiver Operating Characteristic (ROC) curve analysis  The performance of PLR and NLR is shown in Figure 1. Discrimination at the PTE was poor overall (0.5< AUC < 0.6), although there were significant indicators. The area under the curve for PLR was estimated as 0.559 (P-Value:0.001) and for NLR as 0.567 (P-Value<0.001). The threshold for PLR was estimated as 120.5 as the best cut-off point, moreover, the ideal cut-off for the NLR was estimated as 3.5 (Table 4).  3.4. Linear regression analyses for simplified PESI-score determinants in PTE patients  In the linear regression analysis, higher neutrophil count in 24 hours prior to CTA was identified as a potential indicator of PTE severity based on the simplified PESI-score (Beta coefficient: 0.149, P-Value: 0.038) (Table 5) (Figure 2). |
|  |  | *Case-control study—*Report numbers in each exposure category, or summary measures of exposure |  |  |
|  |  | *Cross-sectional study—*Report numbers of outcome events or summary measures |  |  |
| Main results | 16 | (*a*) Give unadjusted estimates and, if applicable, confounder-adjusted estimates and their precision (eg, 95% confidence interval). Make clear which confounders were adjusted for and why they were included | - |  |
|  |  | (*b*) Report category boundaries when continuous variables were categorized | - |  |
|  |  | (*c*) If relevant, consider translating estimates of relative risk into absolute risk for a meaningful time period | - |  |

Continued on next page

| Other analyses | 17 | Report other analyses done—eg analyses of subgroups and interactions, and sensitivity analyses |  |  |
| --- | --- | --- | --- | --- |
| Discussion | | | | |
| Key results | 18 | Summarise key results with reference to study objectives | 8 | Our study indicated that PLR significantly and independently predicts PTE occurrence in hospitalized COVID-19 patients. Furthermore, the bivariate test revealed an association between NLR and PTE incidence in these patients, notwithstanding regression analysis revealed that the correlation was not independently significant. Additionally, a greater neutrophil count was shown to be a marker that is associated with a higher PTE severity (higher simplified-PESI score). |
| Limitations | 19 | Discuss limitations of the study, taking into account sources of potential bias or imprecision. Discuss both direction and magnitude of any potential bias | 10 | There were some limitations inherent to this study. Firstly, the retrospective design of the study was implicated in the development of some biases and hidden confounders. Selection bias might have occurred as the patient data were collected only after a set of requisites became accessible. Secondly, we conducted a monocentric study; consequently, our results are not fully applicable to various COVID-19 patients population. Thirdly, the number of patients included in the study was restricted due to partial loss of information which was inevitable because of the study design. Fourthly, the data on the antiviral and anti-inflammatory medications received by the patients during hospitalization was not available. Therefore, the effect of these drugs on the incidence of PTE and changes in CBC parameters in our COVID-19 patients could not be assessed as confounders. Lastly, any statistics regarding the COVID-19 vaccination of our subjects were not obtainable; thus, this study could not differentiate the effect of SARS-CoV-2 vaccines on the PTE occurrence. |
| Interpretation | 20 | Give a cautious overall interpretation of results considering objectives, limitations, multiplicity of analyses, results from similar studies, and other relevant evidence | 8-10 | 4.1. NLR and PLR predictive value in COVID-19 infection and its pulmonary embolism complication  A systematic review and meta-analysis demonstrated that NLR has adequate predictive values on disease severity and mortality in patients developing COVID-19 infection. In severe or non-survival patients with COVID-19, the lymphocytes count decreases progressively, while the neutrophils count gradually increases (18). This may be due to excessive inflammation and immune suppression caused by SARS-CoV-2 infection. On the one hand, neutrophils are generally regarded as pro-inflammatory cells with a range of antimicrobial activities, which can be triggered by virus-related inflammatory factors, such as interleukin-6 and interleukin-8. On the other hand, systematic inflammation triggered by SARS-CoV-2 significantly depresses cellular immunity, leading to a decrease in CD3 + T cells, CD4 + T cells, and CD8 + T cells. In addition, SARS-CoV-2-infected T cells may also cause cytopathic effects on T cells. Therefore, the ratio of neutrophils to lymphocytes increases (18, 22). Even though the etiopathogenesis of PTE in COVID-19 is not completely understood, factors related to the acute inflammatory response to the disease may be contributing to a dysregulation of the equilibrium of procoagulant and anticoagulant mechanisms. Accordingly, it could reasonably be predicted that higher NLR values were also found in COVID-19 patients with PTE when compared to patients with non-PTE. A Single-center prospective cohort study by Castillejo et al. indicated higher baseline, peak, and prior-to-CTPA NLR values in PTE groups which were statistically significant compared to the non-PTE group (14). This observation was consistent with our bivariate analysis of NLR and PTE incidence; however, regression analysis did not show this significance.  A systematic review and meta-analysis which investigated the prognostic role of PLR in COVID-19 noted that a higher level of PLR on admission in COVID‐19 patients is correlated with increased morbidity and mortality but evidence regarding this issue has low quality (23), but another systematic review and meta-analysis focusing on the association between haematological indices and COVID-19 progression and mortality found that PLR had no significant correlation with progression and fatality of the disease (24). Based on this review of the literature, it can be concluded that NLR is better at predicting the severity and mortality of COVID-19 disease than PLR (25). On the contrary, our study showed PLR but not NLR significantly and independently predicts PTE incidence in hospitalized COVID-19 patients. PLR was shown to be positively correlated with CT pulmonary artery obstruction index which suggests that higher PLR is associated with escalated thrombus burden (26). Consequently, we could postulate that for thrombotic complications of COVID-19 like PTE, PLR has a more precise predictive value.  Considering the cut-off point for the predictive power of NLR and PLR, ROC curve analysis in our study showed a threshold of 3.5 for NLR with 81.4% sensitivity and 29.9% specificity and a threshold of 120.5 for PLR with 76.8% sensitivity and 35.3% specificity. Another observational, analytical, retrospective cohort study demonstrated a threshold of 13.67 for NLR with 67.7% sensitivity and 81% specificity and a threshold of 207.06 with 74.2% sensitivity and 61.3% specificity to predict the occurrence of acute PTE in COVID-19 patients (27). The difference between our cut-off values and the study by Muresan et al. can be ascribed to the various time of laboratory data acquisition. In our study, we utilized laboratory results 24 hours before CTA but Muresan et al. used their first laboratory analyses. Nonetheless, it may be concluded from these results that earlier NLR and PLR calculations, with fairly acceptable specificity for NLR and fairly acceptable sensitivity for PLR in the COVID-19 disease course, could predict the incidence of PTE with higher cut-off values; in comparison, later NLR and PLR calculation could also predict PTE incidence with to some extent acceptable sensitivity but very low specificity with lower cut-off values.  4.2. Association between haematological indices and pulmonary embolism severity (s-PESI score) in COVID-19 patients  The Pulmonary Embolism Severity Index (PESI) was developed to estimate 30-day mortality in patients with acute PTE. The simplified PESI (s-PESI) showed similar prognostic accuracy, clinical utility, and more convenience in use compared with the original PESI (28). An increased s-PESI was shown to be associated with a worse PTE prognosis in COVID-19 patients (29). We found that a higher neutrophil count 24 hours before PTE diagnosis via CTA is correlated with a higher s-PESI score. Hence, we postulate that neutrophil count could be a prognostic factor of PTE severity in COVID-19 patients.  Thoreau et al. study demonstrated that a neutrophil count of more than 7.0 G/L is associated with an increased risk of PTE and also the composite criterion combining a D-dimer level of more than 2000 ng/mL and neutrophils count of more than 7.0 G/L was associated with an increased risk of death, ICU transfer, and longer hospital stay, nevertheless they found PTE occurrence did not affect time to ICU transfer or death, nor did it influence time to hospital discharge (30). Although they observed PTE occurrence does not correlate with worse outcomes, PTE severity as higher neutrophil count could indicate it might have led to a worse prognosis in their patient population which was not assessed in this study.  In another study by Strazzulla et al., the neutrophil count was associated with the diagnosis of acute PTE while no CBC parameters, including neutrophil, were associated with mortality at day seven (15). This disparity between our findings and this study may be attributable to the differing follow-up duration as the s-PESI score estimates 30-day mortality in PTE patients but Strazzulla et al. considered 7-day mortality in their statistical analysis. |
| Generalisability | 21 | Discuss the generalisability (external validity) of the study results | 10 | Among haematological indices, NLR and more precisely PLR are cost-effective and simply calculable markers that can assist physicians in determining whether or not COVID-19 patients with clinically probable PTE require CT angiography and the higher neutrophil count can be employed as an indicator of PTE severity in COVID-19 patients. Further large multicenter and prospective studies are warranted to support these findings and distinguish the effect of SARS-CoV-2 variants, anti-viral and anti-inflammatory medications, and COVID-19 vaccination on the predictive value of CBC parameters for PTE incidence in COVID-19 patients. |
| Other information | |  | | |
| Funding | 22 | Give the source of funding and the role of the funders for the present study and, if applicable, for the original study on which the present article is based | - | Not applicable |

*Give information separately for cases and controls in case-control studies and, if applicable, for exposed and unexposed groups in cohort and cross-sectional studies.

**Note:** An Explanation and Elaboration article discusses each checklist item and gives methodological background and published examples of transparent reporting. The STROBE checklist is best used in conjunction with this article (freely available on the Web sites of PLoS Medicine at http://www.plosmedicine.org/, Annals of Internal Medicine at http://www.annals.org/, and Epidemiology at http://www.epidem.com/). Information on the STROBE Initiative is available at www.strobe-statement.org.
